# ADVANCING NURSING STUDENTS’ DEMENTIA KNOWLEDGE THROUGH ENGAGEMENT WITH PEOPLE LIVING WITH DEMENTIA

**DOI:** 10.1093/geroni/igad104.1577

**Published:** 2023-12-21

## Abstract

The numbers of people living with dementia are overwhelming. Dementia education is important to prepare nursing students to care for this population. To better understand the reality of dementia, we incorporated an expert by experience in teaching a pilot course on dementia. This training was offered to senior nursing students as an elective in their final semester of undergraduate work. Our goal was to teach students about dementia, what it’s like to live with dementia, and actions they can take to support people living with dementia in nursing homes and other settings. The course incorporated clinical experiences at a community memory care facility and small group discussions co-led by a person with dementia and a nursing instructor. Students also participated in an adult day program by leading activities and interacting with people with dementia. Eleven students participated in the pilot course. At program completion, students were invited to share their experiences in a focus group. Qualitative analysis of student feedback identified the benefits of the pilot, including reduced stigma of dementia, increased empathy of those living with dementia, and improved skills in explaining the impact on those diagnosed with dementia. Students appreciated the clinical experiences because it allowed them opportunities to apply their knowledge of dementia and experience the individuality of dementia. This pilot empowered nursing students during their final semester to appreciate, provide care, and support the needs of the people living with dementia.

